# A New Method of Wheelset Bearing Fault Diagnosis

**DOI:** 10.3390/e24101381

**Published:** 2022-09-28

**Authors:** Runtao Sun, Jianwei Yang, Dechen Yao, Jinhai Wang

**Affiliations:** 1 School of Mechanical-Electronic and Vehicle Engineering, Beijing University of Civil Engineering and Architecture, Beijing 100044, China; 2 Beijing Key Laboratory of Performance Guarantee on Urban Rail Transit Vehicles, Beijing University of Civil Engineering and Architecture, Beijing 100044, China; 3 Beijing Engineering Research Center of Monitoring for Construction Safety, Beijing 100044, China

**Keywords:** rolling bearing, compound fault, Ramanujan subspace decomposition, fault feature extraction

## Abstract

During the movement of rail trains, trains are often subjected to harsh operating conditions such as variable speed and heavy loads. It is therefore vital to find a solution for the issue of rolling bearing malfunction diagnostics in such circumstances. This study proposes an adaptive technique for defect identification based on multipoint optimal minimum entropy deconvolution adjusted (MOMEDA) and Ramanujan subspace decomposition. MOMEDA optimally filters the signal and enhances the shock component corresponding to the defect, after which the signal is automatically decomposed into a sequence of signal components using Ramanujan subspace decomposition. The method’s benefit stems from the flawless integration of the two methods and the addition of the adaptable module. It addresses the issues that the conventional signal decomposition and subspace decomposition methods have with redundant parts and significant inaccuracies in fault feature extraction for the vibration signals under loud noise. Finally, it is evaluated through simulation and experimentation in comparison to the current widely used signal decomposition techniques. According to the findings of the envelope spectrum analysis, the novel technique can precisely extract the composite flaws that are present in the bearing, even when there is significant noise interference. Additionally, the signal-to-noise ratio (SNR) and fault defect index were introduced to quantitatively demonstrate the novel method’s denoising and potent fault extraction capabilities, respectively. The approach works well for identifying bearing faults in train wheelsets.

## 1. Introduction

More and more individuals are preferring to use rail transportation as a result of the industry’s rapid development. However, there are potential train malfunctions and safety dangers as a result of the increasing speed of rail trains and long operating hours in a difficult working environment [1,2]. The wheelset bearing of the train, one of the crucial components of the railway travel section, has the dual responsibility of supporting the weight and preserving the wheels’ regular operation. The ease and comfort of train operation will be immediately impacted by its condition, and in extreme circumstances, significant operating accidents will occur. In order to ensure that trains run safely, it is crucial to study the diagnosis of train wheelset bearing issues. Wheelset bearing problems should also be identified quickly and fixed.

When compared to other rotating machinery, the diagnosis of train wheelset bearings is distinct. In the first place, the majority of traditional rolling bearing systems are in a stable and good working environment, and the fault information is evident and less upsetting when fault diagnosis is conducted on them; in contrast, the operating environment for wheelset bearings is highly harsh due to the operational environment of train operation, complex driving circumstances, and the influence between wheels and rails, among other factors. Additionally, they have a wide variety of intricate fault information. It makes fault diagnosis more challenging. Second, the traditional rolling bearing system has minimal noise interference, which results in a small number of fault types and single fault components when a defect needs to be diagnosed. In contrast, the wheel-to-wheel bearing has many fault types and complicated components when a fault arises because of the complex noise influence of the outside environment during train operation. Finding a defect diagnosis method for train wheelset bearings is so crucial [3,4].

Many academics have researched and summarized the various fault diagnosis techniques used in the field of fault diagnosis, as well as the fault diagnosis of various industrial systems. Among these, the study of defect diagnosis based on signal processing techniques is currently receiving a large amount of interest. The is because the system’s fault signal, which carries a wealth of defect information, can be easily gathered by the sensor. The requirements of fault diagnosis can be successfully met by processing them [5,6,7]. Simultaneously, choosing an appropriate approach to extract the fault information from the extremely rich and complex fault information included in the collected fault signals becomes crucial to solving the issue [8,9,10].

Signal decomposition as a class of signal processing method is able to decompose the fault information contained in the signal into different components [11,12,13]. Fault information can be obtained by analyzing the signal components. Empirical mode decomposition (EMD), as one of the most classical methods, has received a large amount of attention and research from scholars. EMD is widely used in the field of fault diagnosis due to its adaptive nature and simple definition of decomposition components [14]. However, there are some problems in the decomposition results. To address the problems of endpoint effect and modal confusion of EMD, scholars have improved EMD and proposed ensemble empirical mode decomposition (EEMD), local characteristic scale decomposition (LCD), variational mode decomposition (VMD), and time-varying filter-based empirical mode decomposition (TVFEMD) and applied them to the field of fault diagnosis well [15,16,17]. EEMD [18] improves the decomposition of EMD by adding Gaussian white noise. LCD [19] improves the decomposition of EMD at signal endpoints by redefining the IMF components. VMD [20] has good robustness and effectively solves the modal aliasing, so it works well for the decomposition of signals containing noise. TVFEMD [21] improves the frequency separation performance and resilience of EMD by creating new time-varying filters and recommending the intrinsic mode function (IMF) bandwidth criterion to improve the separation of different feature information. EFD [22] solves the problem of overlapping decomposition patterns, optimizes the number of signal decompositions, and has a strong immunity to noise. SSD [23] is a decomposition method for narrowband data based on adaptive energy and frequency, and it can effectively analyze nonlinear non-stationary time series with noise. For more complex signal samples, many scholars have introduced deep learning into the mix as well [24,25,26]. Accordingly, the deep learning algorithm is able to perform signal decomposition on more signal samples and extract more fault information.

In recent years, the method of subspace projection in signal decomposition has received much attention. It decomposes the signal into different subspaces by creating mutually independent subspace matrices. Compared with the traditional signal decomposition methods, it can ensure the independence of the decomposed components and the simplicity of the algorithm. It has been applied to the field of fault diagnosis. Xu [27] proposed a fast algorithm for fast subspace decomposition (FSD) for subspace decomposition, which significantly improves the speed of the subspace decomposition algorithm by a novel construction of sample covariance matrix. Molla [28] used the Hilbert spectrum of the signal for subspace decomposition, which effectively improves the effectiveness and efficiency of subspace decomposition, but the robustness in the decomposition process still needs to be improved. Vaidyanathan [29,30] introduced the Ramanujan sum and proposed the Ramanujan cycle transform, which successfully applied the Ramanujan sum to subspace decomposition, but it has certain requirements for the decomposed signal and does not apply to all signals. The above methods have been able to be successfully applied to the field of fault diagnosis, but there are still some difficulties for the diagnosis of bearing faults under strong noise and complex working conditions during the driving process of urban rail trains, such as strong noise interference during decomposition, as well as difficulties in distinguishing fault characteristics when compound faults occur.

This work suggests an adaptive feature extraction method based on MOMEDA and Ramanujan subspace decomposition to address a number of issues with defect diagnostics of train wheelset bearings. First, the MOMEDA technique selects the best pulse deconvolution position and weight of the original signal for filtering, thereby highlighting the impact component of the original signal. After that, we generated the circular matrix according to subspace Ramanujan sum sequence, and the filtered signal is projected to the subspace to obtain the projected components. Finally, to complete the adaptive decomposition process, the residual signal is computed to repeat the projection on the relevant subspace, and the projection termination time is determined on the basis of the energy ratio of the residual signal to the starting signal. In order to observe the fault information hidden in the generated projection components, envelope spectrum analysis was performed concurrently. The outcomes of simulations and experiments demonstrated that the new method that was developed is successful in identifying the composite fault characteristic frequencies of wheelset bearings in a noisy environment.

The following are this paper’s significant contributions:

We propose the use of a new technique for diagnosing wheel pair bearing faults that effectively combine MOMEDA with Ramanujan subspace decomposition. This technique can extract the composite fault characteristic frequencies of bearings in strongly noisy environments.

The projection component of the signal can be obtained adaptively for any signal on the basis of the energy ratio of the residual signal and the starting signal as the termination condition.

We go into great detail about the MOMEDA and Ramanujan subspace decomposition techniques in Section 2. Additionally, in Section 2.4, we present the new method’s basic algorithm and flow. To evaluate the novel method’s capacity to extract faults, we simulated the vibration signal of a rolling bearing with a compound fault, as discussed in Section 3. As discussed in Section 4, we obtained the vibration signal of the wheelset bearing using the train wheelset experimental bench. We compare the new method with the widely used signal processing techniques, and also compare the method’s ability to denoise and extract fault features in greater detail using two indices, SNR and fault defect index. Finally, we summarize the content of this article in Section 5.

## 2. Theoretical Approach

### 2.1. Multi-Point Optimal Minimum Entropy Deconvolution Adjusted (MOMEDA)

In 2016, deconvolution algorithms for detecting multiple pulse locations were proposed by G.L. McDonald [31,32], which can accurately locate the position of each pulse component through a non-iterative computational process and enhance the extracted pulse components, while being able to reduce the amplitude of other irrelevant components of the signal, such as noise. It is the main process.

The multi-D-parameter is used as the objective function in the deconvolution process, and the multi-D-parameter is maximized by studying the values of the filter coefficients, i.e., the optimal deconvolution.

(1) $$\mathrm{MDN}\left(\vec{y},\vec{t}\right)=\frac{1}{∥\vec{t}∥}\frac{{\vec{t}}^{T}\vec{y}}{∥\vec{y}∥}$$

(2) $$\mathrm{MOMEDA}:\mathrm{maxMDN}\left(\vec{y},\vec{t}\right)=\underset{\vec{f}}{max}\frac{{\vec{t}}^{T}\vec{y}}{∥\vec{y}∥}$$

where $\vec{t}$ is the constant target vector used to determine the position and weight of the target pulse components, and ${\vec{t}}^{T}$ represents its transposition; $\vec{y}$ is output signal; and ∥·∥ indicates its parameter number. The problem of finding the maximum value can be solved by the derivative, i.e., taking the derivative of the filter coefficients $\vec{f}$, we obtain Equation (3):

(3) $$\frac{d}{d\vec{f}}\left(\frac{{\vec{t}}^{T}\vec{y}}{∥\vec{y}∥}\right)=\frac{d}{d\vec{f}}\frac{t_{1}y_{1}}{∥\vec{y}∥}+\frac{d}{d\vec{f}}\frac{t_{2}y_{2}}{∥\vec{y}∥}+\ldots +\frac{d}{d\vec{f}}\frac{t_{N-L}y_{N-L}}{∥\vec{y}∥}$$

Of which, $\frac{d}{d\vec{f}}\frac{t_{k}y_{k}}{∥\vec{y}∥}=∥\vec{y}{∥}^{-1}t_{k}{\vec{M}}_{k}-∥\vec{y}{∥}^{-3}t_{k}y_{k}X_{0}\vec{y}$

$${\vec{M}}_{k}=\left[\begin{array}{c} x_{k+L-1}\\ x_{k+L-2}\\ \vdots \\ x_{k} \end{array}\right]$$

where L represents the filter length, N is the number of the input signal x, and $X_{0}$ denotes the matrix of input signal construction. Bringing these into Equation (3) yields, we can obtain Equation (4):

(4) $$\frac{d}{d\vec{f}}\left(\frac{{\vec{t}}^{T}\vec{y}}{∥\vec{y}∥}\right)=∥\vec{y}{∥}^{-1}\left(\begin{array}{c} t_{1}{\vec{M}}_{1}+t_{2}{\vec{M}}_{2}+\ldots \\ +t_{N-L}{\vec{M}}_{N-L} \end{array}\right)-∥\vec{y}{∥}^{-3}{\vec{t}}^{T}\vec{y}X_{0}\vec{y}$$

Simplify Equation (4) by making $t_{1}{\vec{M}}_{1}+t_{2}{\vec{M}}_{2}+\ldots +t_{N-L}{\vec{M}}_{N-L}=X_{0}\vec{t}$, and letting the derivative be 0, we obtain Equation (5):

(5) $$∥\vec{y}{∥}^{-1}X_{0}\vec{t}-∥\vec{y}{∥}^{-3}{\vec{t}}^{T}\vec{y}X_{0}\vec{y}=\vec{0}$$

Continue the simplification of Equation (5) yields, we obtain Equation (6):

(6) $$\frac{{\vec{t}}^{T}\vec{y}}{∥\vec{y}{∥}^{2}}X_{0}\vec{y}=X_{0}\vec{t}$$

where $\vec{y}=X_{0}^{T}\vec{f}$. Moreover, assuming ${\left(X_{0}X_{0}^{T}\right)}^{-1}$ exists, the filter coefficients $\vec{f}$ and the output signal $\vec{y}$ can be obtained as follows:

(7) $$\vec{f}={\left(X_{0}X_{0}^{T}\right)}^{-1}X_{0}\vec{t}$$

(8) $$X_{0}={\left[\begin{array}{ccccc} x_{L} & x_{L+1} & x_{L+2} & \cdots & x_{N}\\ x_{L-1} & x_{L} & x_{L+1} & \cdots & x_{N-1}\\ x_{L-2} & x_{L-1} & x_{L} & \cdots & x_{N-2}\\ \vdots & \vdots & \vdots & \ddots & \vdots \\ x_{1} & x_{2} & x_{3} & \cdots & x_{N-L+1} \end{array}\right]}_{L*\left(N-L+1\right)}$$

(9) $$\vec{y}=X_{0}^{T}\vec{f}$$

### 2.2. Ramanujan Subspace Decomposition

Subspace decomposition, as one of the effective methods in signal decomposition methods, can decompose the signal into parts with different characteristic components in different subspaces, and also can effectively separate the signal from the noise well. Due to the good symmetry and orthogonal nature of Ramanujan and sequence, it can be effectively applied to the construction of subspace and extract the characteristic period of the signal [33,34,35]. The main process of Ramanujan subspace decomposition is as follows.

#### 2.2.1. Ramanujan Sum Sequences and Their Properties

Srinivasa Ramanujan defines a sequence of sums by the triangular summation method:

(10) $$c_{q}\left(n\right)=\overset{q}{\underset{\begin{array}{c} k=1\\ \left(k,q\right)=1 \end{array}}{\sum }}e^{j2\pi kn/q}$$

where q represents the exact period of the sequence; (k,q)=1 denotes the greatest common divisor of k and q is 1, i.e., mutually prime; n represents the number of elements in the sum sequences; and j indicates imaginary units. To transform the sum sequence into the real number field, according to Euler’s formula: $e^{jx}=\cos x+j\sin x$, we obtain

(11) $$c_{q}\left(n\right)=\overset{q}{\underset{\begin{array}{c} k=1\\ \left(k,q\right)=1 \end{array}}{\sum }}\cos\left(2\pi kn/q\right)$$

Thus, the Ramanujan sum sequence can be obtained as a sequence that is symmetric in the real number field and has a period.

#### 2.2.2. Ramanujan Subspace

The Ramanujan subspace can be built from the Ramanujan and sequence $c_{q}\left(0\right)$, and the following integer cyclic matrix $B_{q}$ is introduced first.

(12) $$B_{q}=\left[\begin{array}{ccccc} c_{q}\left(0\right) & c_{q}\left(q-1\right) & c_{q}\left(q-2\right) & \ldots & c_{q}\left(1\right)\\ c_{q}\left(1\right) & c_{q}\left(0\right) & c_{q}\left(q-1\right) & \ldots & c_{q}\left(2\right)\\ c_{q}\left(2\right) & c_{q}\left(1\right) & c_{q}\left(0\right) & \ldots & c_{q}\left(3\right)\\ \vdots & \vdots & \vdots & \vdots & \vdots \\ c_{q}\left(q-2\right) & c_{q}\left(q-3\right) & c_{q}\left(q-4\right) & \ldots & c_{q}\left(q-1\right)\\ c_{q}\left(q-1\right) & c_{q}\left(q-2\right) & c_{q}\left(q-3\right) & \ldots & c_{q}\left(0\right) \end{array}\right]$$

The matrix $B_{q}$ is a Hermitian matrix, and the column space of the matrix $B_{q}$ is defined as the Ramanujan subspace $S_{q}$, so that any signal sequence can be composed of multiple subspaces with different periods q.

Additionally, in order to make the Ramanujan subspace orthogonal to the signal, the orthogonal projection matrix $Z_{q}$ on the subspace $S_{q}$ can be constructed from the matrix $B_{q}$, and the orthogonal projection matrix needs to satisfy $Z_{q}{}^{2}=Z_{q}$, $Z_{q}{}^{T}=Z_{q}$. We obtain,

(13) $$Z_{q}=B_{q}/q$$

Then, for any signal x(n), the projected signal can be expressed as

(14) $$x_{q}=Z_{q}x=B_{q}x/q$$

When given a signal $x\left(n\right)\epsilon R^{N}$ with length N, the projected signal is calculated as follows: the signal x(n) is blocked by the length of the period q, which is divided into M blocks, where M=⎡N/q⌉ represents an integer value taken upwards. x(n) can be expressed as

(15) $$x=\left[\begin{array}{c} x^{\left(1\right)}\\ x^{\left(2\right)}\\ \vdots \\ x^{\left(M\right)} \end{array}\right]$$

Averaging these M blocks of signals yields

(16) $$S=\overset{M}{\underset{m=1}{\sum }}Wx^{\left(m\right)}$$

where *m* denotes the *m*-th block of the taken signal, *s* denotes the mean value of these M blocks of the signal, and W is the diagonal matrix.

(17) $$W=\mathrm{diag}\left(\underset{q-\tilde{N}}{\underbrace{\frac{1}{M},\ldots ,\frac{1}{M}}},\underset{\tilde{N}}{\underbrace{\frac{1}{M-1},\ldots ,\frac{1}{M-1}}}\right)$$

where $\tilde{N}=qM-N$; therefore, according to Equations (14)–(16), the orthogonal projection of the signal x(n) on the subspace $S_{q}$ can be expressed in terms of the chunked mean s.

(18) $$x_{q}=\mathrm{Proj}\left(x,S_{q}\right)=\left[\begin{array}{c} {\bar{x}}_{q}\\ {\bar{x}}_{q}\\ \vdots \\ {\bar{x}}_{q} \end{array}\right]=\left[\begin{array}{c} Z_{q}s\\ Z_{q}s\\ \vdots \\ Z_{q}s \end{array}\right]$$

where $\mathrm{Proj}\left(x,S_{q}\right)$ denotes the projection of the signal x onto the corresponding subspace $S_{q}$.

### 2.3. Signal Periodicity Reference Indicators

In the subspace decomposition process, the choice of period q determines the result of the projection of the signal on the subspace. Therefore, a periodicity reference indicator is introduced to evaluate whether this period is the main part of the decomposition process.

(19) $$P\left(x_{q},q\right)\approx \frac{1}{2q}∥x_{q}{∥}^{2}$$

where $P\left(x_{q},q\right)$ is the periodicity reference indicator, and $∥x_{q}{∥}^{2}$ denotes the energy of the projection corresponding to period q, which can be calculated from the sum of the energy of the current projected signal $∥x_{q}^{*}{∥}^{2}$ minus the energy of the previous lower period.

(20) $$∥x_{q}{∥}^{2}=∥x_{q}^{*}{∥}^{2}-\overset{q-1}{\underset{i=1}{\sum }}∥x_{i}^{*}{∥}^{2}$$

When calculating the periodic reference index according to Equation (17), multiple subspaces need to be created, which increases the complexity of the operation. Therefore, the arithmetic process is simplified by introducing the maximum likelihood estimation as follows:

(21) $$\{\begin{array}{c} ∥x_{q}^{*}{∥}^{2}=\frac{q}{N}\left(\varphi_{x}\left(0\right)+2\overset{M-1}{\underset{l=1}{\sum }}\varphi_{x}\left(lq\right)\right)\\ M=\lfloor N/q\rfloor \; \end{array}$$

where M=⌊N/q⌋ represents the downward rounding function, and $\varphi_{x}$ denotes the autocorrelation function. Therefore, the periodic reference index $P\left(x_{q},q\right)$ of the signal can be obtained according to Equations (18)–(20).

### 2.4. Adaptive Ramanujan Subspace Decomposition Based on MOMEDA

**Figure 1:**
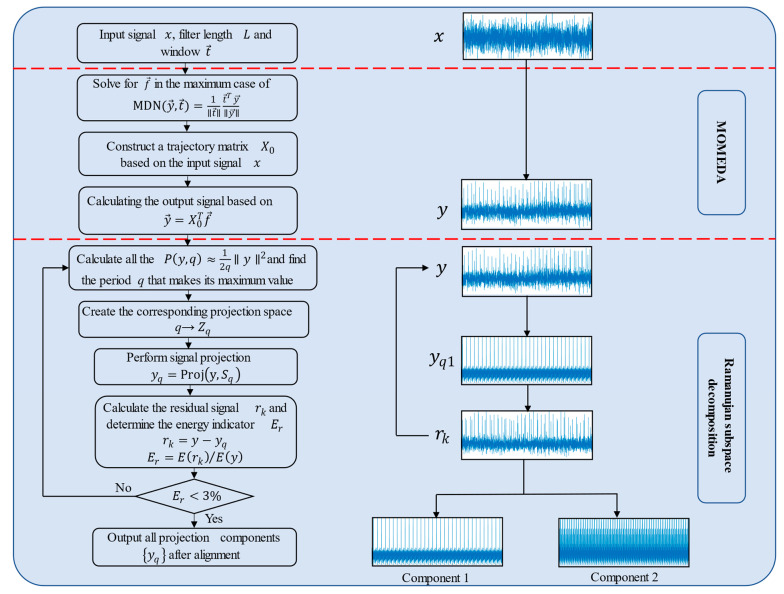
Algorithm flow chart.

Ramanujan subspace decomposition is able to project the signal into a spatial matrix and obtain physically meaningful signal components. However, when the signal has strong noise, the decomposition will become less effective. Moreover, it is not possible to perform adaptive decomposition of the signal. To address these problems, we introduced MOMEDA into the subspace decomposition and iterated the adaptive decomposition by calculating the residual signal. The flow chart of the proposed new method is shown in Figure 1.

In order to implement the adaptive decomposition process of the new method, we designed the iterative approach and the termination conditions concerning the energy ratio, in which the termination condition of the iterative process is judged by judging the ratio of the energy of the residual signal obtained from each iteration to the energy of the signal at the initial entry into the iteration, the energy ratio $E_{r}$, is less than 3% when the iteration ends. For the k-th residual signal $r_{k}$, the signal energy is calculated as follows:

(22) $$\{\begin{array}{l} E_{r}=E\left(r_{k}\right)/E\left(x_{1}\right)<3\%\\ E\left(x\right)=∥x{∥}_{2}^{2} \end{array}$$

where E(x) represents the energy of the signal x, and $∥\cdot {∥}_{2}$ represents the two-parameter number.

The detailed steps of the algorithm are as follows:

Step 1: Select the appropriate MOMEDA filter length and filter window for optimal filtering of the input raw signal according to the input signal length.

Step 2. Construct the corresponding Ramanujan subspace for the processed signal and calculate the projection of the signal on the subspace to obtain the signal components.

Step 3. Calculate the residual signal and its corresponding Ramanujan subspace for circular decomposition and determine whether to continue the decomposition according to the energy index.

Step 4. Arrange the projected signal components according to their frequencies to obtain the final signal decomposition results. Then, perform envelope spectrum analysis on signal components.

## 3. Simulation Experiments

Using the method proposed in this paper, we demonstrated the validity of the method for detecting rolling bearing faults under strong noise and compound fault conditions. We established a simulation signal under faults affecting the inner and outer rings of rolling bearings, and the expression is shown in Equation (22), where $x_{1}\left(t\right)$ and $x_{2}\left(t\right)$ represent the vibration signal under the fault of the outer ring and inner ring of the rolling bearing, respectively; n(t) represents the noise, taking the signal-to-noise ratio equal to −15dB; $f_{n1}$ and $f_{n2}$ represent the resonant frequency, $f_{n1}=3000$ and $f_{n2}=2500$, where the outer ring fault frequency is set to $f_{1}=1/T_{1}=90\mathrm{Hz}$, and the inner ring fault frequency is set to $f_{2}=1/T_{2}=134\mathrm{Hz}$; A represents the amplitude, A=5; and g represents the damping coefficient, g=0.1; sampling frequency: $f_{s}=8192$, sampling points: N=4096.

(23) $$\{\begin{array}{l} x\left(t\right)=x_{1}\left(t\right)+x_{2}\left(t\right)+n\left(t\right)\\ x_{1}\left(t\right)=\sum_{i}As_{1}\left(t-iT_{1}\right)\\ x_{2}\left(t\right)=\sum_{i}As_{2}\left(t-iT_{2}\right)\\ s_{1}\left(t\right)=e^{-2\pi gf_{n1}t}\sin\left(2\pi f_{n1}\sqrt{1-g^{2}}t\right)\\ s_{2}\left(t\right)=e^{-2\pi gf_{n2}t}\sin\left(2\pi f_{n2}\sqrt{1-g^{2}}t\right) \end{array}$$

**Figure 2:**
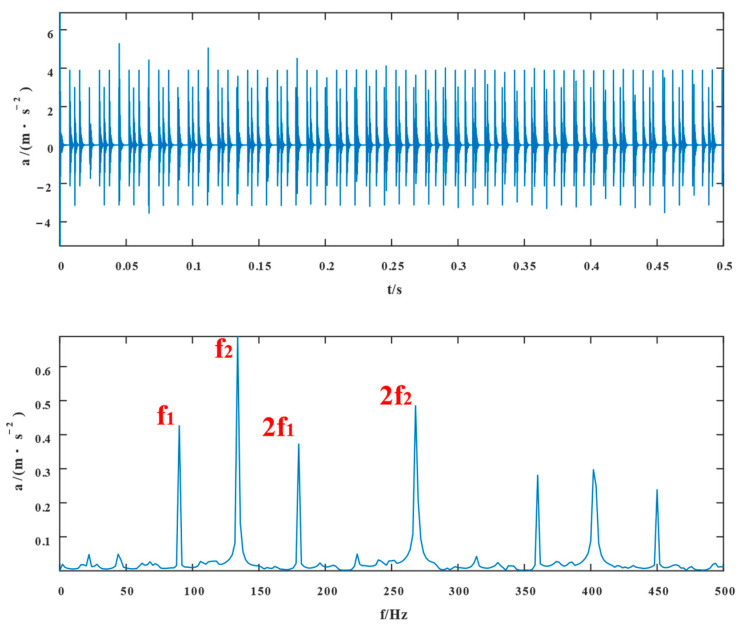
Time domain waveform of the simulated signal without adding noise and its spectral envelope in the frequency domain.

Figure 2 depicts the time-domain diagrams and envelope spectrums of composite fault signals in the inner and outer rings of a bearing without noise, respectively. In Figure 2, the simulated signal shows evidence of a periodic shock component. The envelope spectrum indicates that the inner and outer ring fault frequencies and their multiplication frequencies were included in the simulated signal.

**Figure 3:**
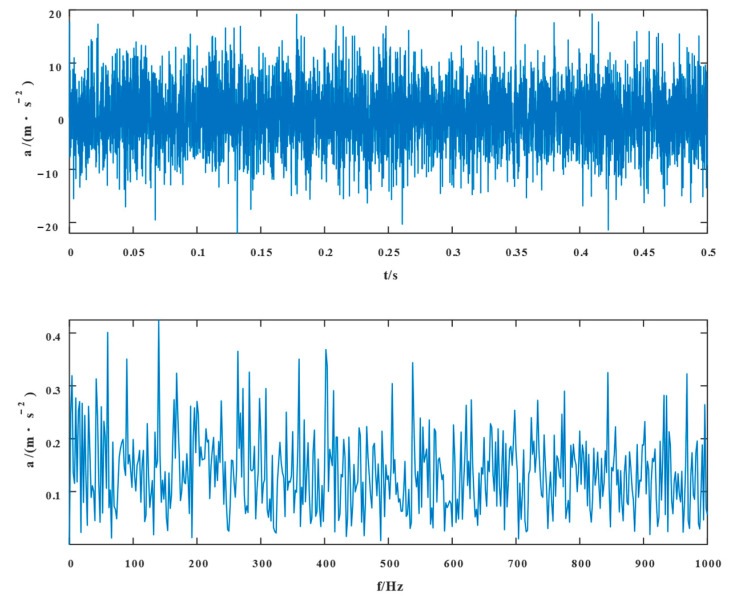
Waveform of the simulated signal in the time domain and its envelope spectrum in the frequency domain after adding noise.

Figure 3 shows the time domain waveforms and the envelope spectrum of the simulated signal. The periodic shock was submerged in strong noise; its envelope spectrum was also severely affected by the noise, and faults within and outside the rings were not distinguished by their characteristic frequencies.

The proposed method was compared with current signal decomposition and feature extraction techniques, including variational mode decomposition (VMD), singular spectrum decomposition (SSD), and time-varying filter-based empirical mode decomposition (TVFEMD) by observing the envelope spectra of key elements of each method and examining their noise immunity and ability to extract fault features.

**Figure 4:**
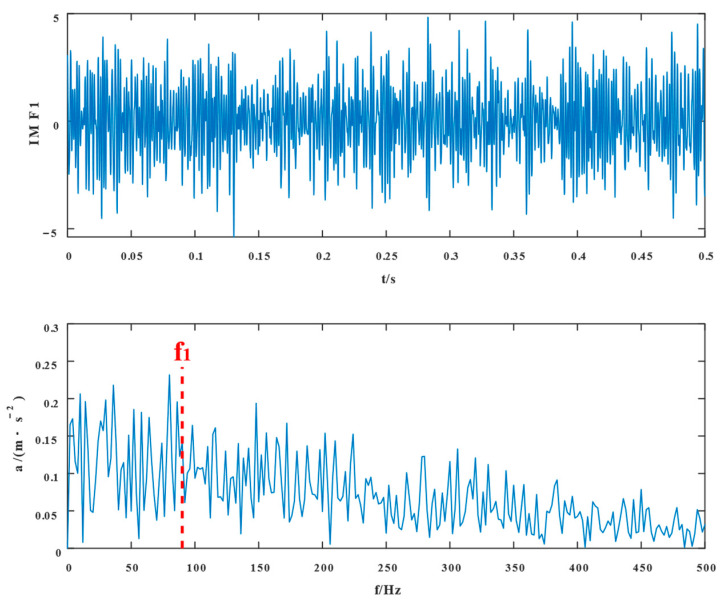
IMF1 and its envelope spectrum of VMD.

**Figure 5:**
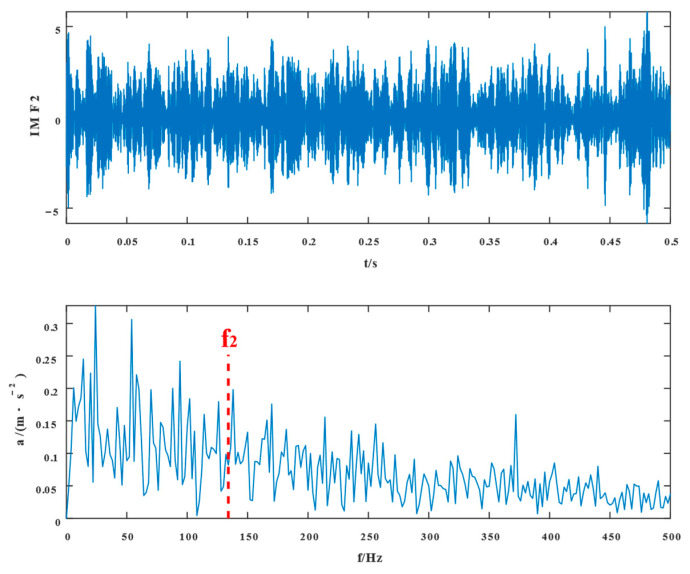
IMF2 and its envelope spectrum of VMD.

To begin with, VMD decomposes the simulated fault signal, and the number of decomposition modes K=2, penalty coefficients α=2000*,* and convergence tolerance $tol=10^{-7}$ were selected in VMD. Figure 4 and Figure 5 show the time domain waveforms of the VMD decomposition components IMF1 and IMF2 and their envelope spectra, respectively.

**Figure 6:**
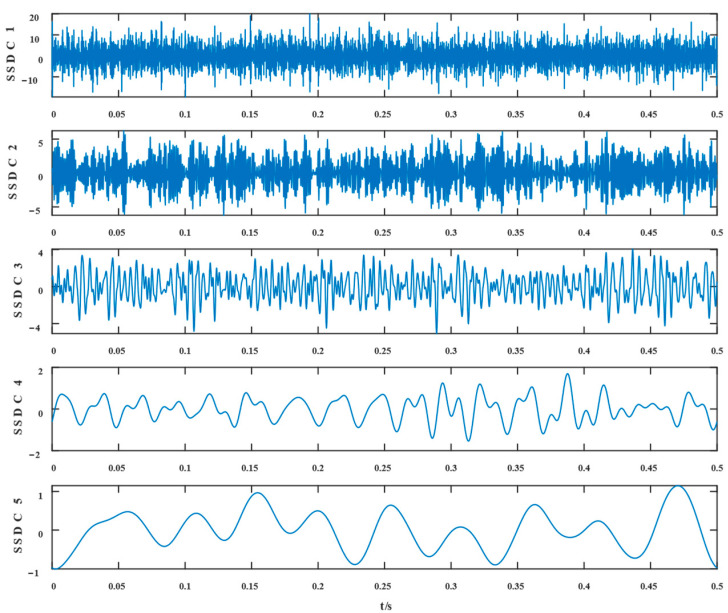
Waveform diagram of the processing result of the simulated signal by SSD.

**Figure 7:**
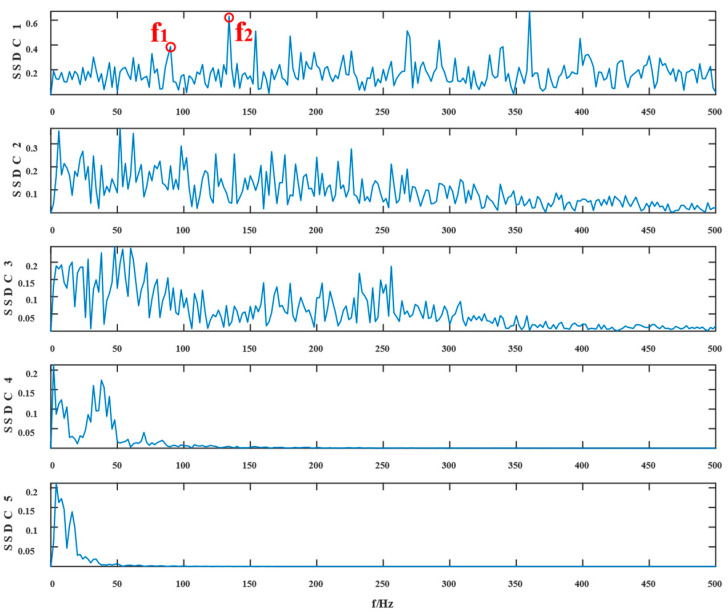
The resultant envelope spectrum of the SSD processing of the simulated signal.

**Figure 8:**
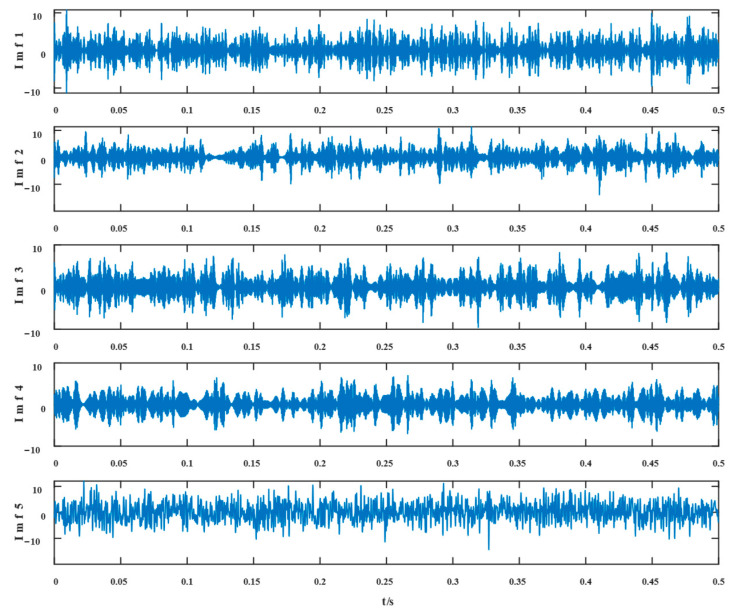
Waveform diagram of the processing result of the simulated signal by TVFEMD.

**Figure 9:**
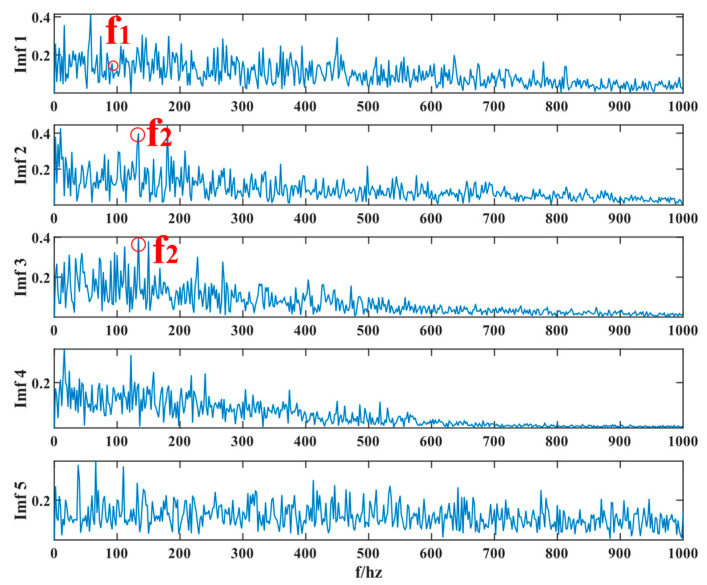
The resultant envelope spectrum of the TVFEMD processing of the simulated signal.

Similarly, the simulated signals were processed by SSD and TVFEMD methods to obtain the decomposition results and their envelope spectra shown in Figure 6, Figure 7, Figure 8 and Figure 9.

**Figure 10:**
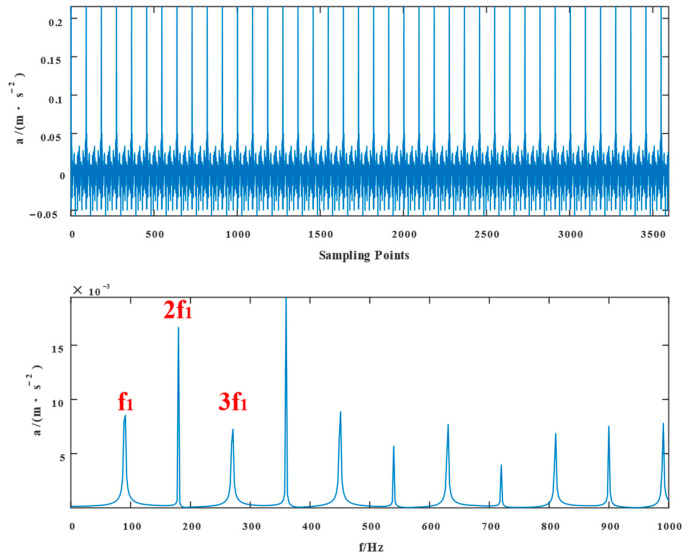
Time domain waveform of the first signal component and its envelope spectrum.

**Figure 11:**
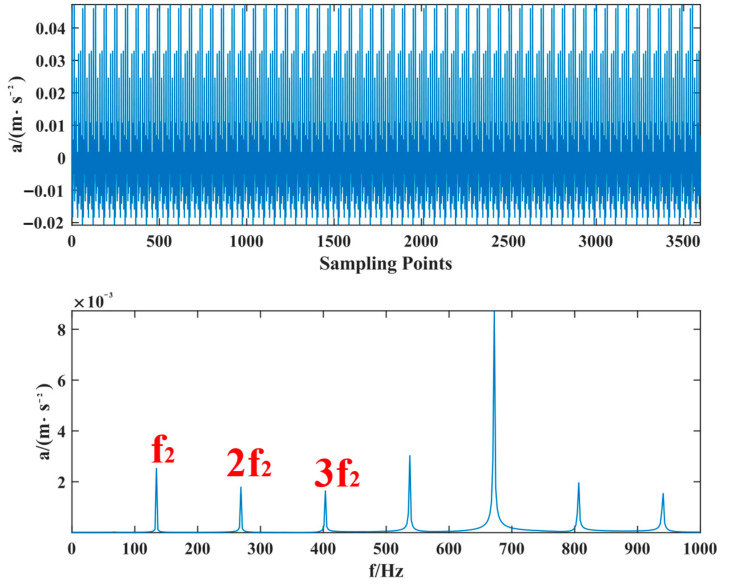
Time domain waveform of the second signal component and its envelope spectrum.

The proposed method was compared with VMD, SSD, and TVFEMD to confirm its superiority and efficacy in handling the composite faults shown in Figure 4, Figure 5, Figure 6, Figure 7, Figure 8, Figure 9, Figure 10 and Figure 11. Through the analysis of time domain diagram, VMD can see the periodic vibration shock, but still the interference of noise is strong in the strong noise environment; SSD and TVFEMD effectively separated the different components of the signal, and further analysis was needed. According to the envelope spectrum analysis in the frequency domain, VMD could not distinguish the composite fault characteristic frequency; SSD highlighted the fault frequency but did not distinguish it; TVFEMD distinguished it, but the problem of modal mixing appeared. The decomposition results of the new method are shown in Figure 10 and Figure 11. The first signal component’s time domain waveform and envelope spectrum are shown in Figure 10, where the outer loop fault frequency and its multiplier can be seen; Figure 11 illustrates the second signal component’s time domain waveform and envelope spectrum. The envelope spectrum also revealed the inner loop fault frequency and its multiplier. This method’s ability to extract the complex fault features of bearings’ inner and outer rings under severe conditions was thus confirmed.

To further highlight the noise immunity and the ability to extract fault features of the new method, the signal-to-noise ratio (SNR) and the fault defect index of the signal components after decomposition by various methods were calculated.

According to the literature [36], the following equation can be used to determine the SNR:

(24) $$\mathrm{SNR}=10\log10(\frac{\sum_{i=1}^{N}x^{2}(i)}{\sum_{i=1}^{N}{\left(x(i)-\hat{x}(i)\right)}^{2}})$$

where, separately, x(i):i=1,2,…,N displays the original signal and $\hat{x}\left(i\right)$ denotes the decomposed signal. The average value was used to determine the method’s overall SNR after computing the SNR for each component of the signal. Table 1 compares the SNR of several methods and highlights the new method’s higher noise immunity and noise reduction.

**Figure 12:**
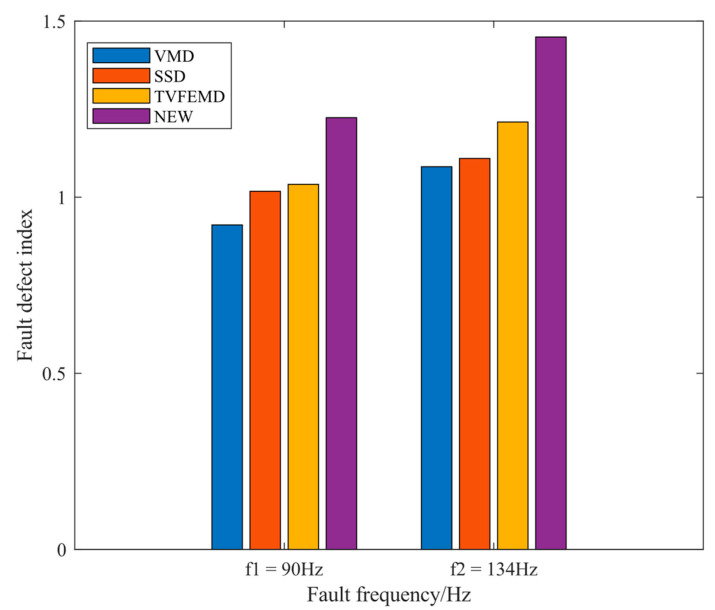
Fault defect index result graph for the four methods.

A fault defect index was introduced for assessment in order to further contrast the effectiveness of various methods for the extraction of fault features. According to the literature, [37,38] provides the formula for calculating the fault defect index (FDI).

(25) $$\alpha \left(f\right)=\frac{\mathrm{mean}\left(A\left(f-1\right),A\left(f+1\right)\right)}{\mathrm{mean}\left(A\left(f-10\right),A\left(f+10\right)\right)}$$

(26) $$\bar{\alpha }=\frac{\alpha \left(f\right)+\alpha \left(2f\right)+\alpha \left(3f\right)}{3}$$

where α(f) is the fault defect index at the corresponding frequency, A(f) is the envelope spectrum amplitude of the relevant frequency, and f denotes the fault frequency. Considering the error in the actual calculation of the fault frequency of rolling bearings, the mean value of f±1 was chosen as the envelope spectrum amplitude at the fault frequency f. $\bar{\alpha }$ represents the mean value of the fault defect index, which is obtained by finding the mean value of the fault defect index at 1–3 times the frequency of the fault. The proposed novel method can be examined to better emphasize the frequency of fault features in light of the fault defect index result in Figure 12, which shows this.

## 4. Experimental Analysis

We developed a model of the vibration signal of the wheel pair rolling bearing in the event of a compound defect through simulation experiments, and the benefits of the suggested approach were well illustrated by comparison tests and two evaluation indices. However, the simulations alone cannot show the good results of the method for the vibration signal during the actual operation of the wheel. The vibration signals measured in the experiments may contain a variety of influences that are not present in the simulated signals. In order to assess the bearing vibration signals of moving train wheels in a real environment and further confirm the efficacy and benefits of the technology, we put up a wheel pair simulation test bench.

**Figure 13:**
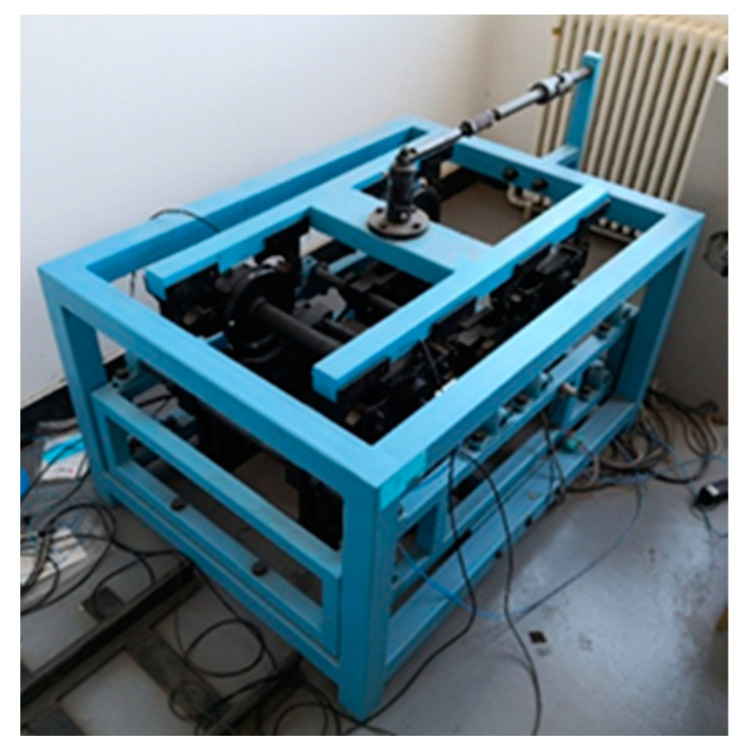
Bearing test stand.

**Figure 14:**
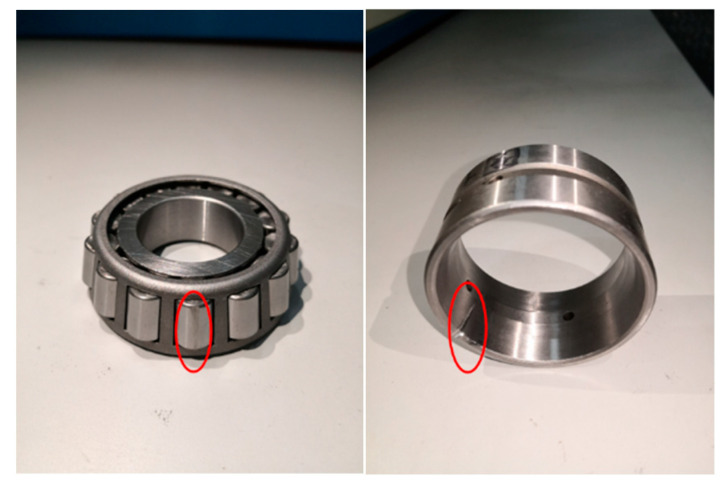
Bearing compound fault setting ((**left**): rolling element fault; (**right**): outer ring fault).

In order to more deeply study the effectiveness of the rolling bearing for the feature extraction of compound faults under strong noise, the experiment adopted the rolling bearing simulation experiment bench of the train wheel pair shown in Figure 13 to conduct the experiment of compound faults of rolling bearing. The test stand was built on the basis of a real train wheel pair and scaled down, consisting of the train wheel pair, drive motor, wheel track, axle box, and console. In order to simulate the real operating conditions of a rail train in the laboratory, the wheels and tracks were processed by material removal methods to simulate the condition of the tracks and wheels in real conditions. The wheelset bearings were also machined simultaneously to mimic the incidence of compound failures. As shown in Figure 14, the defects were added to the rolling element and outer ring, correspondingly. The bearing model HRB 351306 was used, and the main parameters were as follows: number of rolling elements n=14, rolling element diameter d=12mm, bearing pitch diameter D=56mm, rolling element contact angle $\varphi =10^{{}^{\circ}}$. In the experiment, the wheel pair speed 30 km/h and spindle speed were set at 766.8 r/min. We selected the rolling bearing with both outer ring failure and rolling body failure to simulate the compound fault, and the fault setting is shown in Figure 14. According to the bearing outer ring and rolling body fault, the characteristic frequency calculation formula can be obtained, rolling body fault frequency: $f_{g}=31.15\mathrm{Hz}$, and outer ring fault frequency: $f_{w}=70.83\mathrm{Hz}$.

**Figure 15:**
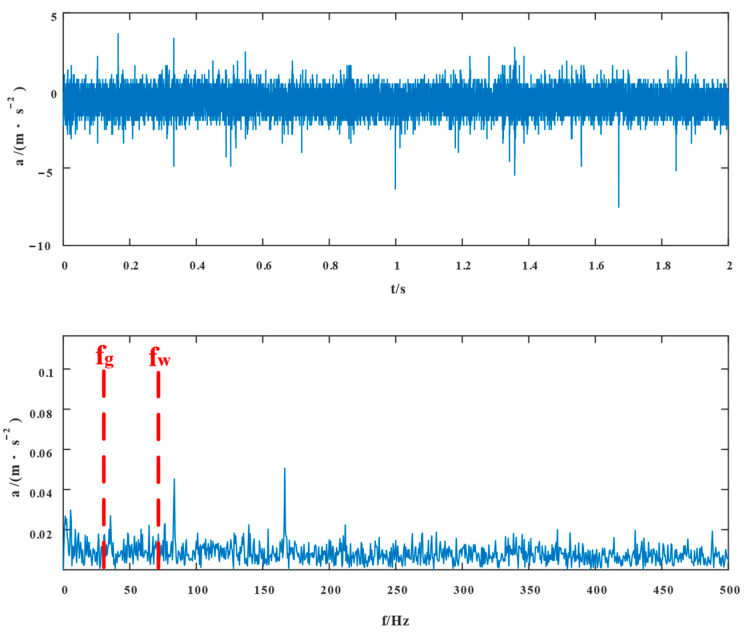
Compound failure signal’s time domain diagram and envelope spectrum.

Figure 15 displays the fault signal’s time domain structure and envelope spectrum, in which the sampling frequency 10,000Hz was 1.5–3.5 s. The time domain diagram shows the existence of shocks, indicating the existence of bearing faults. We cannot accurately extract the characteristic frequency in the fault signal from the envelope spectrum of outer ring and rolling body fault frequencies because it is difficult to see the type of fault frequency from the envelope spectrum alone.

**Figure 16:**
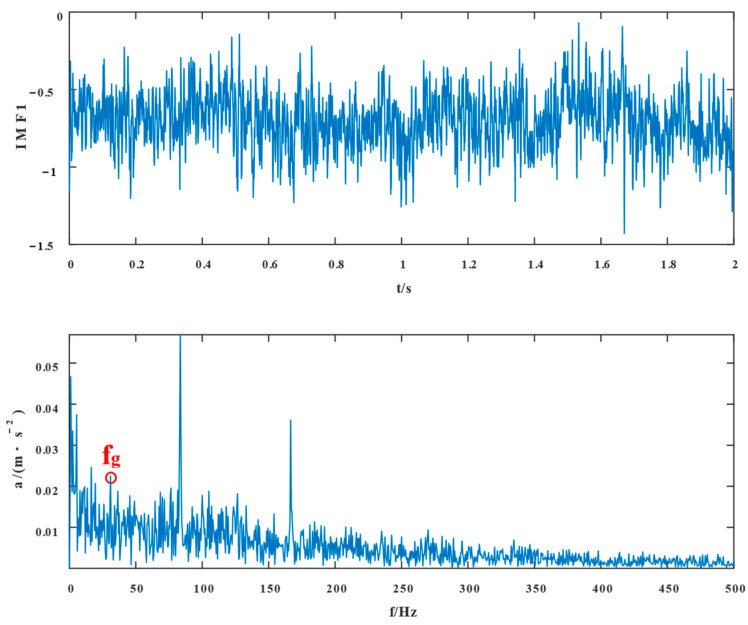
IMF1 and its envelope spectrum of VMD.

**Figure 17:**
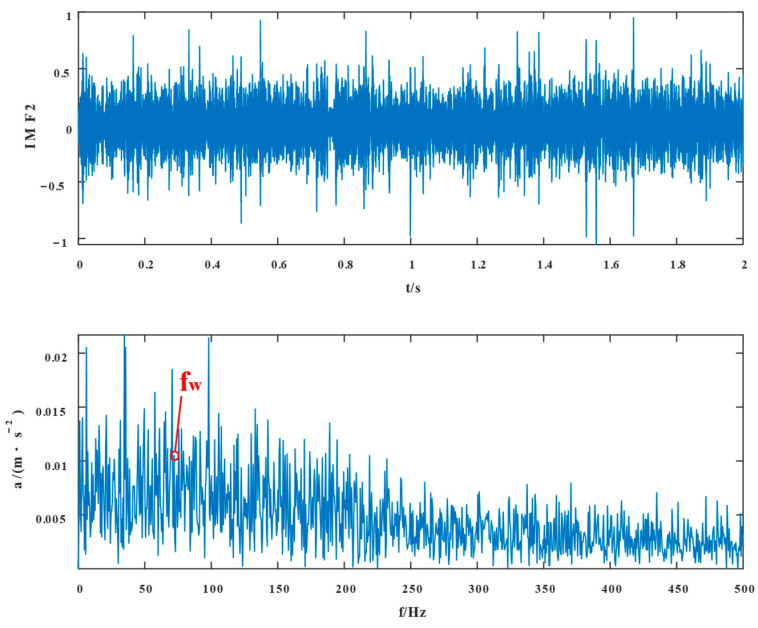
IMF2 and its envelope spectrum of VMD.

**Figure 18:**
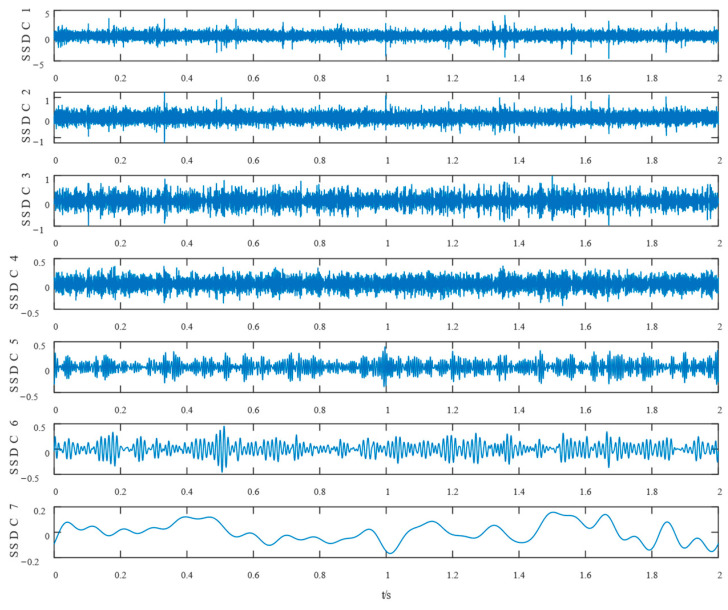
Waveform representation of the simulated signal by SSD.

**Figure 19:**
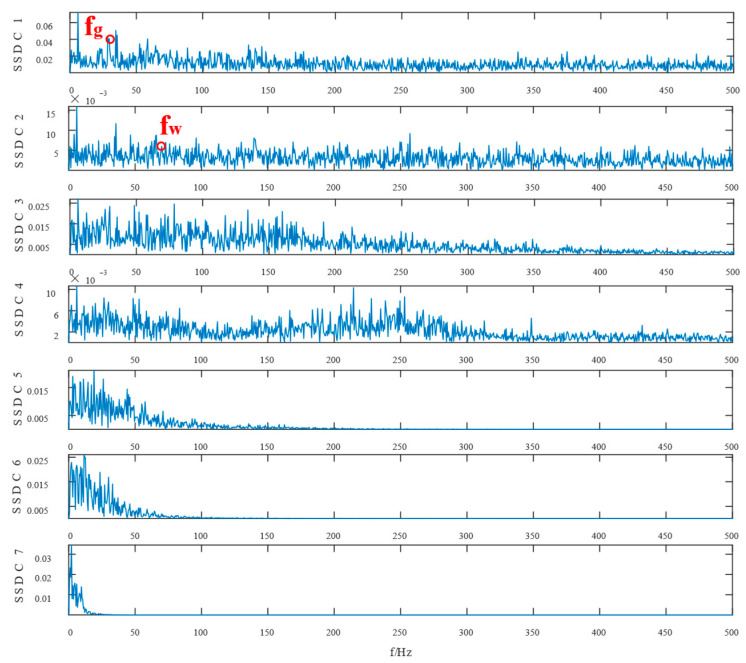
The resulting envelope spectrum of the simulated signal’s SSD processing.

**Figure 20:**
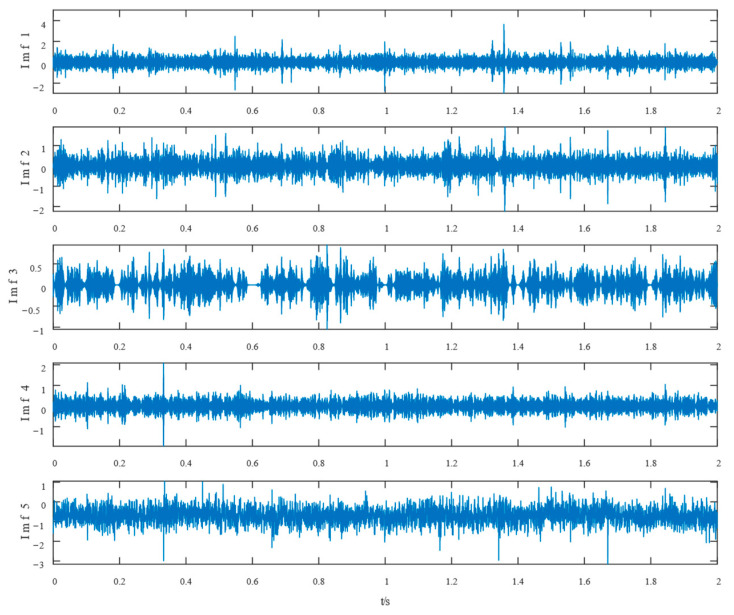
Waveform representation of the simulated signal by TVFEMD.

**Figure 21:**
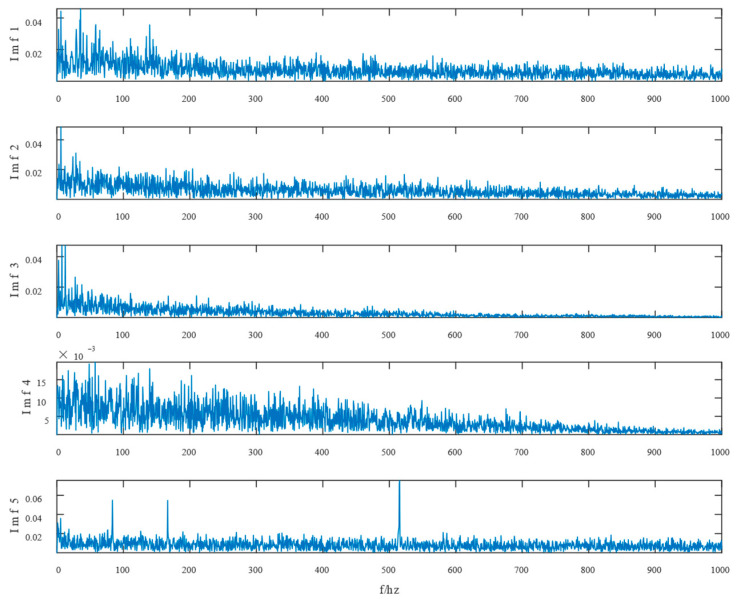
The envelope spectrum of the simulated signal’s TVFEMD processing.

**Figure 22:**
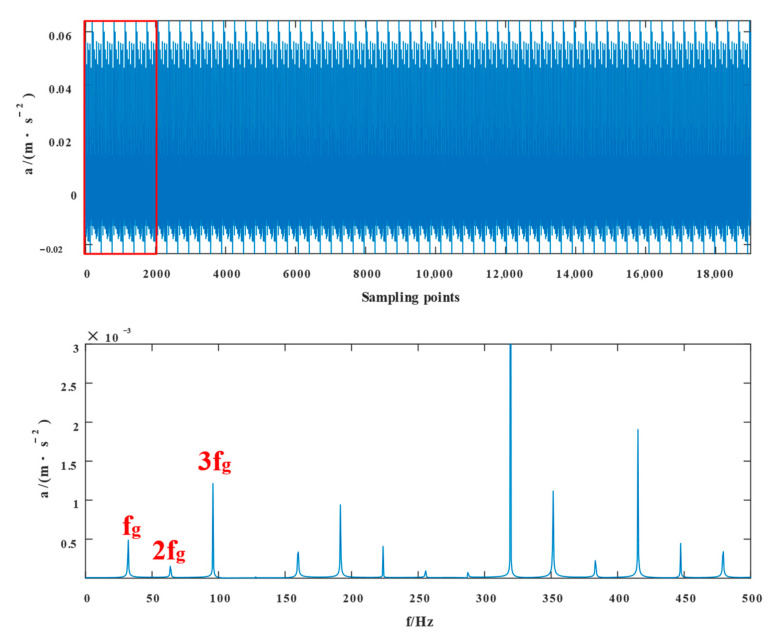
Component 1 time domain diagram and envelope spectrum and its partial enlargement of the new method.

**Figure 23:**
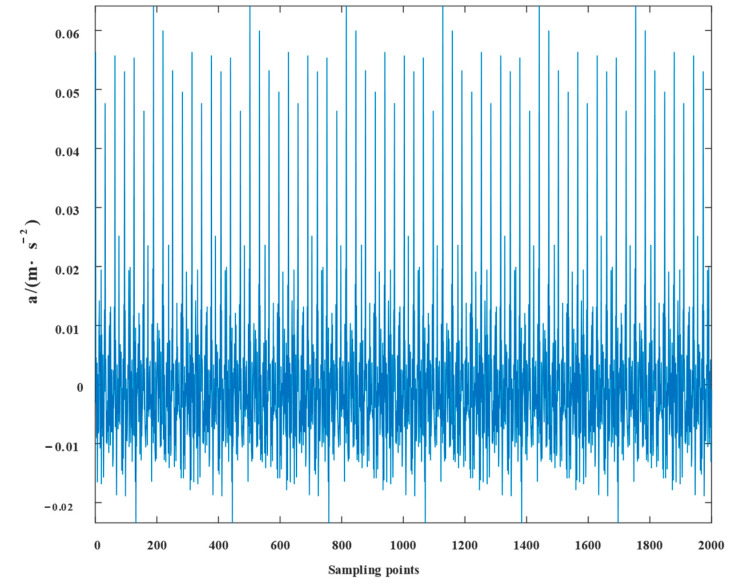
The partial enlargement of the Component 1 time domain diagram.

**Figure 24:**
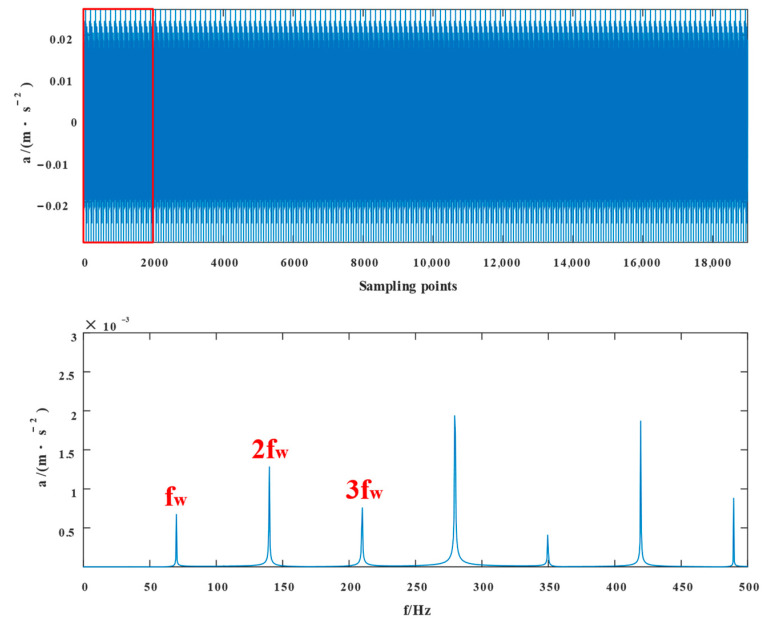
Component 2 time domain diagram and envelope spectrum and its partial enlargement of the new method.

**Figure 25:**
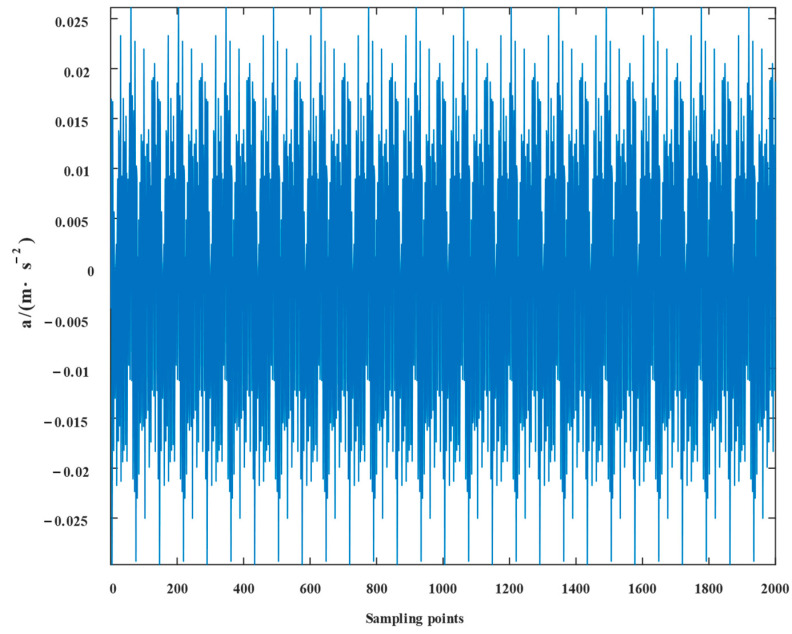
The partial enlargement of the Component 2 time domain diagram.

The experimental waveforms and envelope spectra for VMD, SSD, TVFEMD, and the proposed technique are displayed in Figure 16, Figure 17, Figure 18, Figure 19, Figure 20, Figure 21, Figure 22, Figure 23, Figure 24 and Figure 25. Firstly, the time domain waveform plots were observed, and both VMD and SSD were able to reveal the signal components that contain cycles and shocks; TVFEMD was affected by noise, and only the presence of shocks can be observed; and the proposed new method was able to clearly reveal the periodic shock components of the signal. Then, the envelope spectrum analysis was performed in the frequency domain, and VMD, SSD, and TVFEMD were unable to distinguish and extract the composite fault frequencies. The new method, however, extracts the composite fault characteristics well, and the multiplicative components of the fault frequencies can be clearly observed.

**Figure 26:**
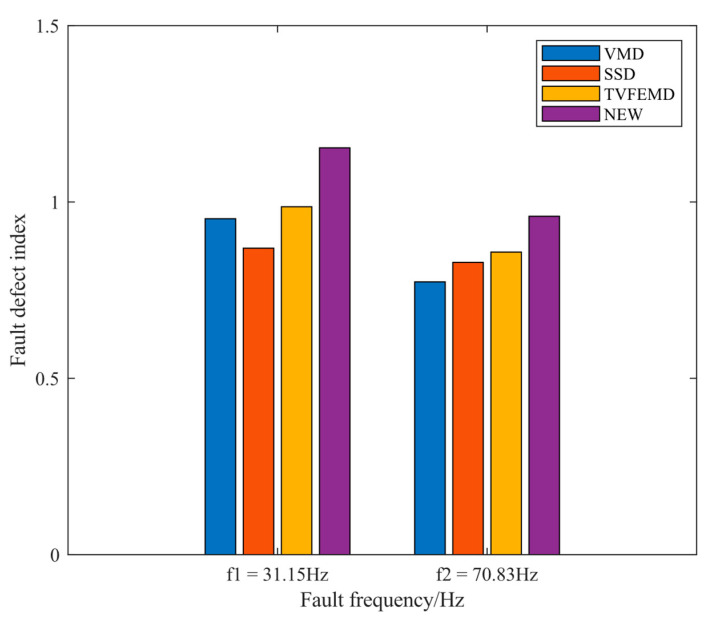
Fault defect index result graph for four methods.

The experimental signals were analyzed for SNR and fault defect index, as shown in Table 2 and Figure 26, to further explore the new method’s capacity to extract fault frequencies and suppress noise. The findings show that the proposed method decomposed the signal with greater SNR and FDI, which improved denoising and noise reduction impact as well as fault extraction capability.

## 5. Conclusions

In this study, rolling bearing fault signals of train wheel pairs in complex settings were detected and analyzed. A method based on MOMEDA and Ramanujan subspace decomposition was then proposed for the fault diagnosis of rolling bearings in this situation, and the results are as follows:

(1) The periodic shock component due to the fault can be accurately extracted and enhanced for the signal affected by environmental noise.
(2) It can effectively decompose the signal into periodic components with different energies according to the periodic reference index to distinguish the parts with different fault characteristics and can effectively reduce the influence of noise.
(3) Compared with VMD, SSD, and TVFEMD, the decomposed components have more obvious fault characteristics, which can be effectively applied to the extraction of fault feature frequencies under strong noise and the presence of compound faults in rolling bearings, providing an effective method for feature extraction in the process of wheelset rolling bearing fault diagnosis.

## Figures and Tables

**Table 1:** Comparison the SNR results of these several methods.

Algorithms	SNR
VMD	0.1275
SSD	−0.9458
TVFEMD	−0.8765
NEW	1.3235

**Table 2:** Comparison the SNR results of these methods.

Algorithms	SNR
VMD	1.1057
SSD	0.4089
TVFEMD	0.7475
NEW	1.7923

## Data Availability

Not applicable.
